# Supplementary material for: A Global Screen for Assembly State Changes of the Mitotic Proteome by SEC-SWATH-MS
Source: Cell Syst. 2020 Feb 26;10(2):133–155.e6. doi: 10.1016/j.cels.2020.01.001 (PMC7042714; doi:10.1016/j.cels.2020.01.001)

O95347 | SMC2_HUMAN | SMC2 CAPE SMC2L1 PRO0324
Monomer MW [kDa]: 135.656 Monomer expected elution fraction: 38

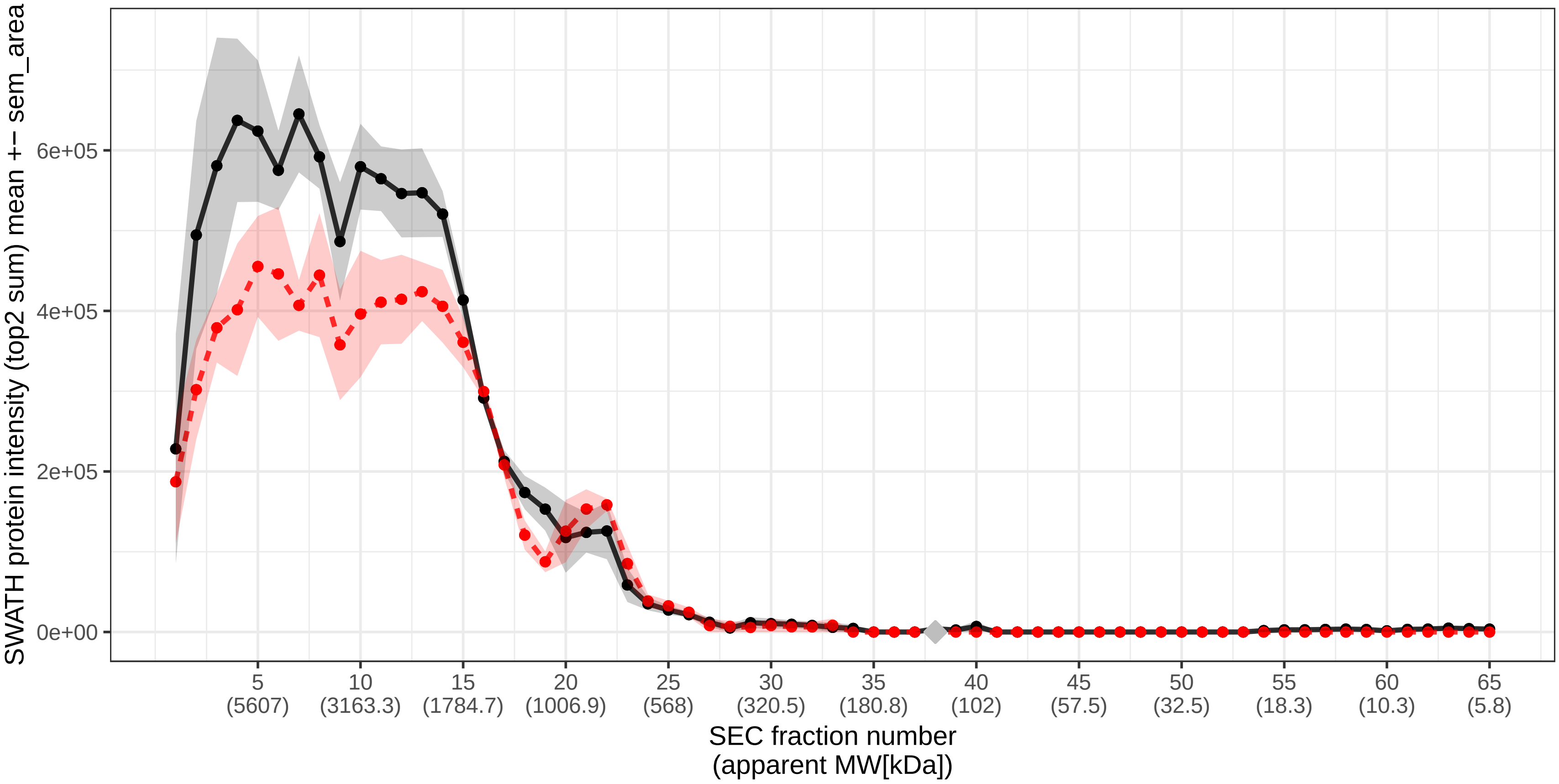

Supplement: Data S1. SEC-SWATH-MS Protein Chromatograms, Related to Figure 1 [file mmc6.zip › SECchrom_O95347_SMC2_HUMAN_SMC2_CAPE_SMC2L1_PRO0324.pdf]
